# Experimentally validated oxidative stress -associated prognostic signatures describe the immune landscape and predict the drug response and prognosis of SKCM

**DOI:** 10.3389/fimmu.2024.1387316

**Published:** 2024-04-10

**Authors:** Dongyun Rong, Yushen Su, Dechao Jia, Zhirui Zeng, Yan Yang, Dalong Wei, Honguan Lu, Yu Cao

**Affiliations:** ^1^Clinical Medical School, Guizhou Medical University, Guiyang, Guizhou, China; ^2^Department of anorectal surgery, Affiliated Hospital of Guizhou Medical University, Guiyang, Guizhou, China; ^3^School of Basic Medicine, Guizhou Medical University, Guiyang, Guizhou, China; ^4^Department of Internal Medicine, The Third Affiliated Hospital of Guizhou Medical University, Duyun, Guizhou, China; ^5^Department of Burns, Plastic Surgery and Wound Repair, Affiliated Hospital of Youjiang Medical University for Nationalities, Baise, Guangxi, China; ^6^Key Laboratory of Tumor Molecular Pathology of Baise, Affiliated Hospital of Youjiang Medical University for Nationalities, Baise, Guangxi, China

**Keywords:** SKCM, oxidative stress network, immunotherapy response, drug resistance, immune

## Abstract

**Background:**

Skin Cutaneous Melanoma (SKCM) incidence is continually increasing, with chemotherapy and immunotherapy being among the most common cancer treatment modalities. This study aims to identify novel biomarkers for chemotherapy and immunotherapy response in SKCM and explore their association with oxidative stress.

**Methods:**

Utilizing TCGA-SKCM RNA-seq data, we employed Weighted Gene Co-expression Network Analysis (WGCNA) and Protein-Protein Interaction (PPI) networks to identify six core genes. Gene co-expression analysis and immune-related analysis were conducted, and specific markers associated with oxidative stress were identified using Gene Set Variation Analysis (GSVA). Single-cell analysis revealed the expression patterns of Oxidative Stress-Associated Genes (OSAG) in the tumor microenvironment. TIDE analysis was employed to explore the association between immune therapy response and OSAG, while CIBERSORT was used to analyze the tumor immune microenvironment. The BEST database demonstrated the impact of the Oxidative Stress signaling pathway on chemotherapy drug resistance. Immunohistochemical staining and ROC curve evaluation were performed to assess the protein expression levels of core genes in SKCM and normal samples, with survival analysis utilized to determine their diagnostic value.

**Results:**

We identified six central genes associated with SKCM metastasis, among which the expression of DSC2 and DSC3 involved in the oxidative stress pathway was closely related to immune cell infiltration. DSC2 influenced drug resistance in SKMC patients. Furthermore, downregulation of DSC2 and DSC3 expression enhanced the response of SKCM patients to immunotherapy.

**Conclusion:**

This study identified two Oxidative Stress-Associated genes as novel biomarkers for SKCM. Additionally, targeting the oxidative stress pathway may serve as a new strategy in clinical practice to enhance SKCM chemotherapy and sensitivity.

## 1 Introduction

Melanoma originates from neuroectodermal melanin-producing cells, known as SKCM, distributed across various tissues, with predilection for skin and mucous membranes. The rising incidence of melanoma is attributed to factors like UV exposure, oxidative stress, DNA damage, and mutations, culminating in its genesis (1). SKCM, characterized by its high incidence, metastasis, and mortality rates, represents the most invasive malignancy of skin melanocytes. Its poor prognosis stems from early metastasis and challenging early-stage diagnosis (2). Given its significant morbidity and economic burden, early detection and stratification of novel biomarkers are imperative for enhancing SKCM management.

Notably, various factors contribute significantly to SKCM development, warranting the identification of biomarkers. For instance, Wang et al. correlated SRGN expression with SKCM and SKCM-metastasis patient survival (3). Zhang et al. associated lower GBP2 expression with reduced immune cell infiltration and poorer SKCM prognosis (4). Additionally, B cell characteristics play pivotal prognostic (5, 6), and predictive roles in SKCM, shaping its immunobiology and potential immunogenomics features (7).

Oxidative stress, implicated in aging, inflammation, and chronic diseases, results from the imbalance between reactive oxygen and nitrogen species and a compromised antioxidant defense system. Studies by Elena Piskounova et al. revealed that oxidative stress inhibits distant metastasis in human melanoma cells (8, 9). Moreover, oxidative stress is linked to various cancers, with drugs like doxorubicin modulating oxidative stress to improve survival (10). Intracellular oxidative stress amplification is explored as a synergistic cascade cancer therapy strategy (11). Recent research developed an oxidative stress-related prognostic model for SKCM, offering new insights for melanoma analysis (12).

Challenges in malignant melanoma diagnosis and treatment include the development of primary or secondary drug resistance in over half of patients, rendering existing treatments ineffective. Addressing this necessitates identifying new molecular therapeutic targets to enhance SKCM patient management. Thus, this article aims to identify and evaluate novel potential biomarkers, offering valuable insights for further exploration.

## 2 Materials and methods

### 2.1 Data collection and processing

Clinical specimens were provided by Guizhou Medical University Affiliated Hospital (Guiyang, China), comprising 92 pairs of tumor and adjacent samples (1-2 cm from tumor tissue). These samples encompassed 24 trunk subtype samples and 68 other subtype samples. Approval for this study was obtained from the Human Characteristics Ethics Committee of Guizhou Medical University, and the principles of the Helsinki Declaration were strictly followed. Informed consent was also obtained from patients providing samples.

Transcriptomic data and related clinical information were sourced from the TCGA database (https://portal.gdc.cancer.gov/projects/TCGA-SKCM). The RNA-sequencing dataset encompasses 472 samples, including 78 samples from Trunk Cutaneous Melanoma (TCM) and 394 samples from other melanoma subtypes. A threshold of 140, post-normalization, was applied to identify outliers using the hierarchical clustering algorithm. The cut-off for FPKM was established at 0.5 to exclude genes with low expression levels. A threshold of 0.5 was set during gene selection. Following merge and batch normalization, differential expression genes (DEGs) are identified using ‘‘sva’’ R package (13–15). Moreover, DEGs were discerned between tumor and normal tissues using a cut-off value of |Log2 fold-change (FC)| > 0.5 and an adjusted *P*-value < 0.05 (16).

### 2.2 Gene co-expression network construction and identification of crucial clinical modules

Genes were analyzed for the construction of a scale-free network via the ‘‘WGCNA’’ methodology (17, 18). This expression network, predicated on weighted gene co-expression data, was established utilizing the top quartile of genes exhibiting the highest variance. Initially, an expression data-derived similarity matrix was generated to compute the absolute values of Pearson correlation coefficients between gene pairs. Subsequently, this similarity matrix was transformed into an adjacency matrix, with the application of a soft threshold that accentuated strong connections while diminishing the significance of weaker correlations within the matrix. This adjacency matrix was further converted into a topological overlap matrix (TOM), designed to more accurately depict the strength of relationships and the degree of connectivity among genes within the dataset. Serving as a measure for assessing network connectivity among genes, TOM was employed as an input for hierarchical clustering analysis. The ‘‘DynamicTreeCut’’ algorithm, utilized within the ‘‘WGCNA’’ R package, facilitated the identification of network modules (19). Following module identification, gene significance (GS) was evaluated, correlating these genes with other biological information. The higher the GS value, the more substantial the prognostic relevance for patients. Hence, an analysis employing Pearson’s correlation coefficient was conducted to ascertain the correlation between identified modules and clinical features, including patient age and cancer stage. Through this approach, ‘‘WGCNA’’ aids in elucidating the connections between gene expression patterns and clinical characteristics, offering vital insights for the discovery of biomarkers and the formulation of therapeutic strategies for disease management.

### 2.3 GO and KEGG enrichment

GO and KEGG enrichment analyses were conducted using the R package ‘‘clusterProfiler’’ to explore pivotal modules, with *P* < 0.05 defined as significant enrichment (20). These analyses aimed at identifying potential signaling pathways, elucidating the biological processes and pathways crucial in the occurrence and progression of melanoma (21).

### 2.4 Construction of PPI network and gene co-expression analysis

PPI network was constructed using the online STRING database (https://string-db.org/). During network construction, disconnected nodes were removed to ensure network connectivity. Subsequently, the network graph of key modules was created using Cytoscape 3.72, revealing crucial interactions by identifying the top 30 ranked genes. Each node in the PPI network represents a protein encoded by a gene, while edges depict interactions between two proteins. To explore the potential of DSC2 and DSC3 as novel targets for regulatory intervention, an analysis was conducted to identify genes that show co-expression with these markers. This analysis highlighted twelve genes exhibiting the highest co-expression coefficients with DSC2 and DSC3. The findings, represented in a circular graph, underscore the importance of further investigation into these associated genes, suggesting they warrant closer scrutiny.

### 2.5 Survival analysis

Survival analysis was conducted using the ‘‘survival’’ and ‘‘survminer’’ packages in R language (22). Initially, patients were stratified into high and low expression groups based on the median gene expression levels, and the association between these gene expression levels and prognosis was determined using the Log-rank test.

### 2.6 Single-cell analysis combined with CIBERSORT examination of immune infiltration

Subsequently, exploration of subpopulation clustering in SKCM at the single-cell level was carried out utilizing the TISCH2 single-cell database (23). Briefly, the ‘‘Seurat’’ package is employed for scrutinizing scRNA-seq data of SKCM (18). Initially, following the exclusion of cells expressing fewer than 250 or exceeding 6000 genes, a logarithmic normalization is performed on gene expression. Subsequently, employing the functions ‘‘FindNeighbors’’ and ‘‘FindClusters’’, individual cells are clustered into distinct subgroups. To further elucidate the roles of DSC2 and DSC3 in immune infiltration, the ‘‘CIBERSORT’’ algorithm was employed to calculate immune infiltration scores in each sample (24). Samples were dichotomized based on the median gene expression levels, comparing the infiltration of various types of immune cells between these two groups and assessing DSC2 and DSC3 respectively. The correlation analysis between gene expression and immune cell infiltration was visualized using the ‘‘ggplot2’’ package, including box plots and correlation scatter plots. Finally, the correlation between genes and immune checkpoints was analyzed using the ‘‘corrplot’’ package and visualized through heat maps. Furthermore, specific gene associations with immune checkpoint genes were evaluated.

### 2.7 Evaluation of immunotherapy response and chemotherapy resistance

In recent years, immunotherapy has emerged as a promising anti-tumor strategy (25), demonstrating favorable anti-tumor effects in SKCM treatment. Hence, further investigation into the role of oxidative stress-related genes in SKCM immunotherapy was conducted. The association between immunotherapy response and oxidative stress-related genes was explored using TIDE analysis. Additionally, the BEST database illustrated the impact of oxidative stress signaling pathways on chemotherapy drug resistance. Immunohistochemical staining and ROC curve analysis were employed to evaluate the protein expression levels of core genes in SKCM and normal samples, and their diagnostic value was determined through survival analysis (26).

### 2.8 Gene set variation analysis

GSVA is a non-parametric unsupervised analytical method used to assess the enrichment of gene sets in transcriptome data (27). The score of oxidative stress were determined by the ‘‘ssGSEA’’ using ‘‘GSVA’’ R package, and the certain signatures that respond to oxidative stress were obtained from database (http://www.informatics.jax.org/vocab/gene_ontology/GO:0006979). This method transforms gene expression data into an expression matrix characterized by a specific set of genes (28). Besides, Pearson correlation analysis was conducted to validate the correlation between hub genes and oxidative stress scores. Through this process, we could assess association between screened genes and oxidative stress.

### 2.9 Immunohistochemistry experiment

In an ambient laboratory environment, tissues were subjected to a 4% Paraformaldehyde fixation process. Subsequently, these specimens were encased within paraffin blocks and dissected into sections of 4 µm in thickness. The process of removing paraffin was conducted utilizing xylene at a temperature of 60°C, succeeded by a graded rehydration sequence employing ethanol concentrations of 100%, 80%, 60%, and 40%. To inhibit the intrinsic peroxidase reactions present in the samples, 3% H_2_O_2_ was utilized, while sodium served the purpose of facilitating antigen retrieval. Following a 16-hour incubation period at 4°C in a medium containing 5% bovine serum albumin, the sections underwent a further incubation with an array of primary antibodies (Santa Cruz, CA, USA), specifically (DSC2; 1:200), (DSC3; 1:200), (DSG1; 1:200), (KRT6B; 1:200), (PKP1; 1:200), and (PKP3; 1:400). Subsequent to this, for a duration of 2 hours at an ambient temperature, the sections were exposed to secondary antibodies targeted against mouse and rabbit immunoglobulins. Visual documentation of the samples was achieved using a high-resolution light microscopy technique, employing magnification levels of x200 and x400. Following this, a staining protocol involving 3,3’ diaminobenzidine and hematoxylin was applied for a minute at ambient temperature. The evaluation of the staining intensity for the genes of interest was performed, with scores assigned as follows: 0 indicating no staining, 1 for weak positivity, 2 for moderate positivity, and 3 for strong positivity, facilitating the quantification of protein expression. The Image Pro Plus software was employed to determine the scoring based on the proportion of positively stained cells, with a scoring rubric of 0-2 for low expression, 3-4 for moderate expression, and 5-6 denoting high expression. *P*-value < 0.05 was interpreted as indicative of a statistically significant variance.

### 2.10 Statistical analysis

All statistical analyses were conducted using packages in the R programming language (version 3.6.3). Prior to statistical analysis, normality tests were performed to determine the appropriate statistical methods. Analysis of normal and non-normal data was conducted using unpaired Student’s t-test and Wilcoxon test, respectively (29). Pearson method was employed for correlation analysis, with *P* < 0.05 considered statistically significant. * *P* < 0.05, ** *P* < 0.01, *** *P* < 0.001, **** *P* < 0.0001.

## 3 Results

### 3.1 Construction of WGCNA co-expression network

**Figure 1:**
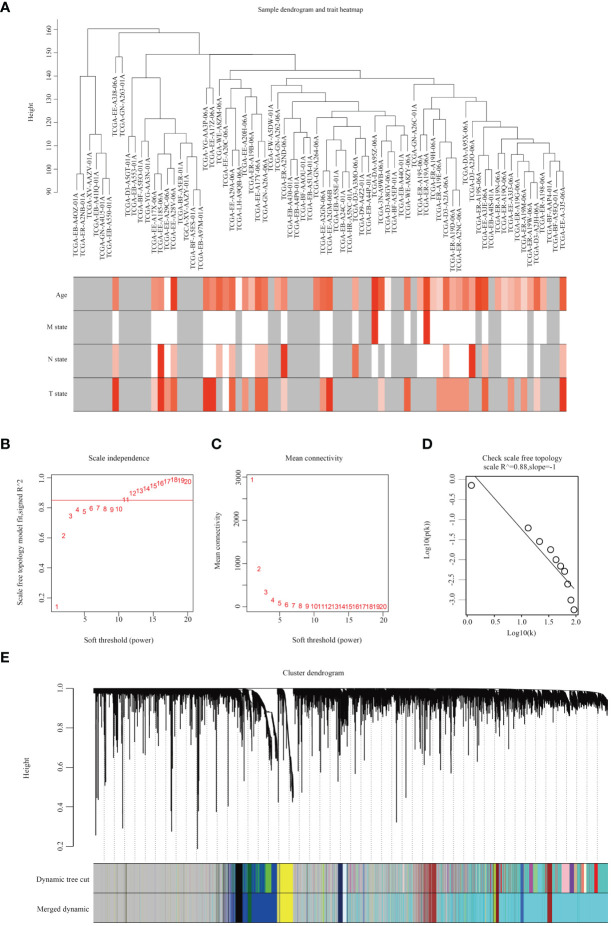
Analysis of SKCM weighted gene co-expression networks by WGCNA. **(A)** Dendrogram illustrating samples of SKCM along with clinical patient characteristics. **(B)** Evaluation of scale independence fit indexes through soft thresholding power analysis. **(C)** Soft thresholding power analysis depicting mean connectivity. **(D)** Demonstration of topology with scale-free scaling at β=11. **(E)** Identification of 12 gene co-expression modules utilizing a dissimilarity measure.

Upon downloading and integrating expression data along with clinical data, no aberrant samples were identified through the sample dendrogram, and most clinical features were documented (**Figure 1A**). Subsequently, the WGCNA network was constructed using the gene expression profile after filtering out low-expressed genes. The WGCNA algorithm was applied to construct co-expression networks and modules for 472 samples from TCGA. We selected the top 25% mutated genes from the TCGA-SKCM cohort and performed clustering analysis using the ‘WGCNA’ R package. With a soft threshold β set to 11, the scale independence of the topological network exceeded 0.85, and the average connectivity approached 0 (**Figures 1B, C**). Therefore, setting the soft threshold to β=11 satisfied the scale-free topology criterion, with R^2 = ^0.88 for the TOM (**Figure 1D**). The dynamic tree cutting algorithm based on TOM was employed to cluster all selected genes, resulting in the division of the tree into 12 modules (**Figure 1E**), each labeled with distinct colors.

### 3.2 Identification of key modules and central genes associated with clinical features

**Figure 2:**
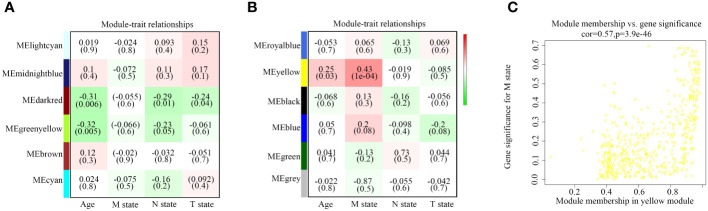
Relationship between modules and traits. **(A, B)** Correlation between modules and age, M state, N state, and T state. **(C)** Comparison of module membership and gene significance highlighted in yellow.

Subsequently, we summarized the co-expression of feature genes and calculated the correlation between feature genes and clinical characteristics. Pathological diagnosis typically includes information on pathological stages M, N, and T at the time of diagnosis. Each module consists of distinct gene clusters, marked in an overlapping heatmap with different colors; red indicating positive correlation and green indicating negative correlation (**Figure 2A**). Among the co-expression modules and features we analyzed, we observed that the yellow module is most closely related to pathological stage M (R=0.43, *P*=1e-04); additionally, this module is positively correlated with age (R=0.25, *P* < 0.05) (**Figure 2B**). Therefore, we consider the yellow module containing 520 genes as a critical gene module and employ it for further analysis (**Figure 2C**).

### 3.3 Analysis of KEGG pathways and GO enrichment

**Figure 3:**
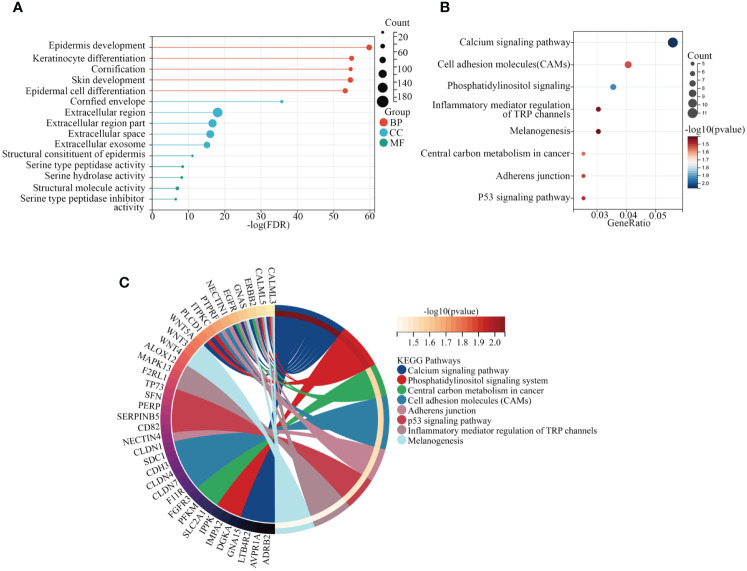
Exploration of KEGG and GO pathways. **(A)** GO analysis of core genes within modules. **(B, C)** KEGG pathway analysis of core genes within modules.

To elucidate the functional mechanisms of the yellow module, we conducted enrichment analyses of GO terms and KEGG pathways. In this study, we initially examined biological processes (BP), molecular functions (MF), and cellular components (CC) to identify the most enriched GO pathways. The results revealed that, in terms of biological processes, these genes are primarily involved in epidermal development, keratinocyte differentiation, and keratinization processes. Regarding cellular components, the main enrichment was observed in cornified envelope, extracellular region, and extracellular region part. Molecular function analysis indicated that these genes are predominantly enriched in epidermal structural components (**Figure 3A**). Subsequently, based on the enrichment results of KEGG pathways, we identified pathways closely associated with tumorigenesis, including central carbon metabolism, cell adhesion molecules, junctional protein binding, and melanogenesis, providing important clues for understanding the functional mechanisms of genes within the yellow module (**Figures 3B, C**).

### 3.4 Relationship between central genes and oxidative stress characteristics

**Figure 4:**
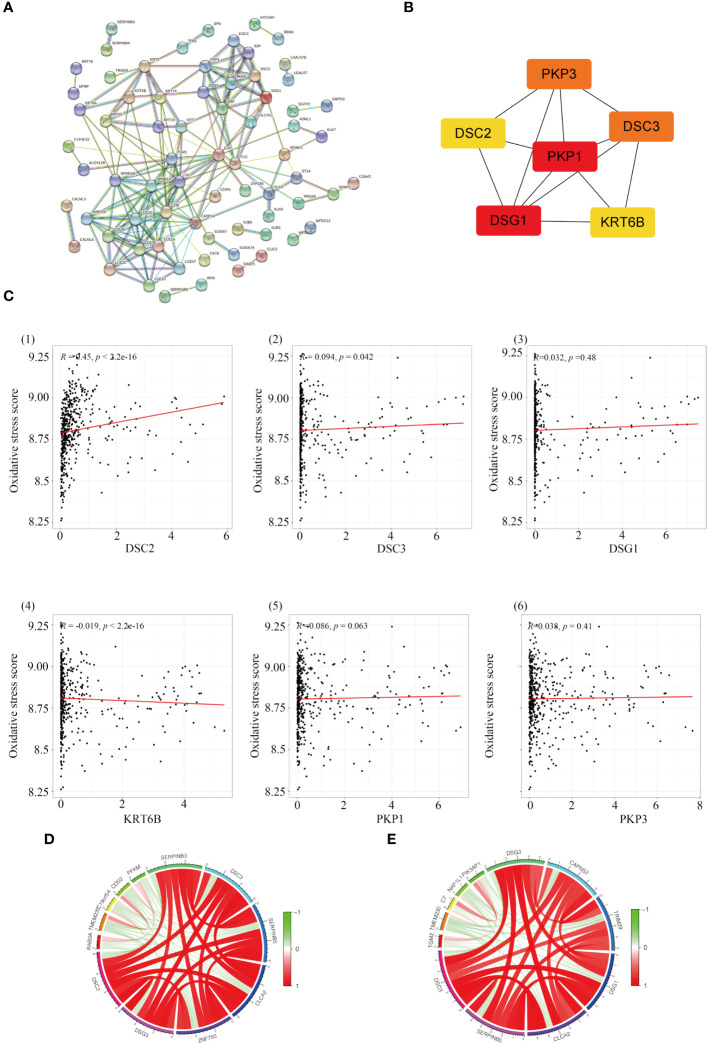
Identification of hub genes in melanoma. **(A)** Construction of a protein-protein interaction network using modules containing core genes. **(B)** Recognition of top 6 node genes (DSC2, DSC3, DSG1, KRT6B, PKP1, PKP3) as key genes for further analysis. **(C)** Investigation into the relationship between gene expression and oxidative stress in SKCM patients. **(D)** Discovery of 12 genes predominantly correlated with DCS2 and the interconnectedness among them. **(E)** Examination of correlation with DCS3 and the interconnectedness among these genes.

To explore the interrelations among genes and identify central genes, an initial analysis was conducted on genes within the yellow module, followed by visualization using STRING. After eliminating unconnected nodes, a PPI network was obtained (**Figure 4A**). Subsequently, employing ‘Cytoscape’, topological parameters for all nodes were computed, and the six highest-ranking node genes were identified as pivotal for subsequent analyses (**Figure 4B**). To investigate the association between central genes (DSC2, DSC3, DSG1, KRT6B, PKP1, and PKP3) and the response to oxidative stress, we employed GSVA to compute the oxidative stress scores for each sample. Pearson correlation analysis revealed a significant correlation between oxidative stress scores and DSC2 (R=0.45; *P* < 0.001) as well as DSC3 (R=0.094; *P* < 0.05), whereas other genes showed no involvement in the oxidative stress response (**Figure 4C**). This underscores the need for further exploration into the relationship between oxidative stress and DSC2, DSC3.

### 3.5 Gene co-expression analysis and survival analysis

**Figure 5:**
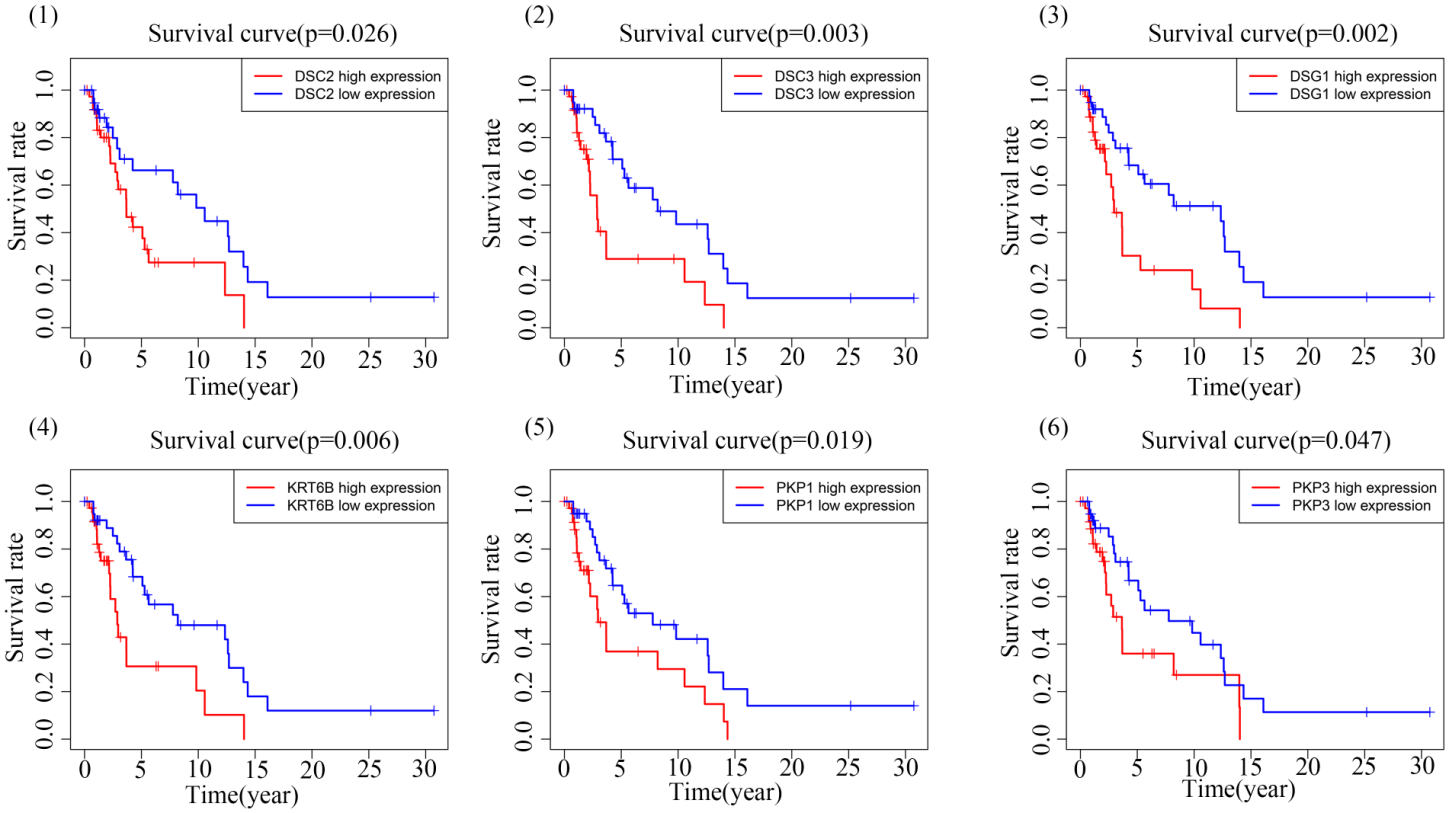
Kaplan-Meier survival plot.

Through correlation analysis, we identified a set of 12 genes highly correlated with both DSC2 and DSC3, and delved into the interrelationships among them. Circular diagrams were presented for DSC2 (**Figure 4D**) and DSC3 (**Figure 4E**) separately to illustrate their associations. KM survival analysis was conducted on these selected central genes, revealing a significant association with the prognosis of SKCM patients (*P* < 0.05). As the expression levels of DSC2, DSC3, DSG1, KRT6B, PKP1, and PKP3 increased, the overall survival time of trunk subtype melanoma patients significantly decreased (**Figure 5**).

### 3.6 Immune infiltration

**Figure 6:**
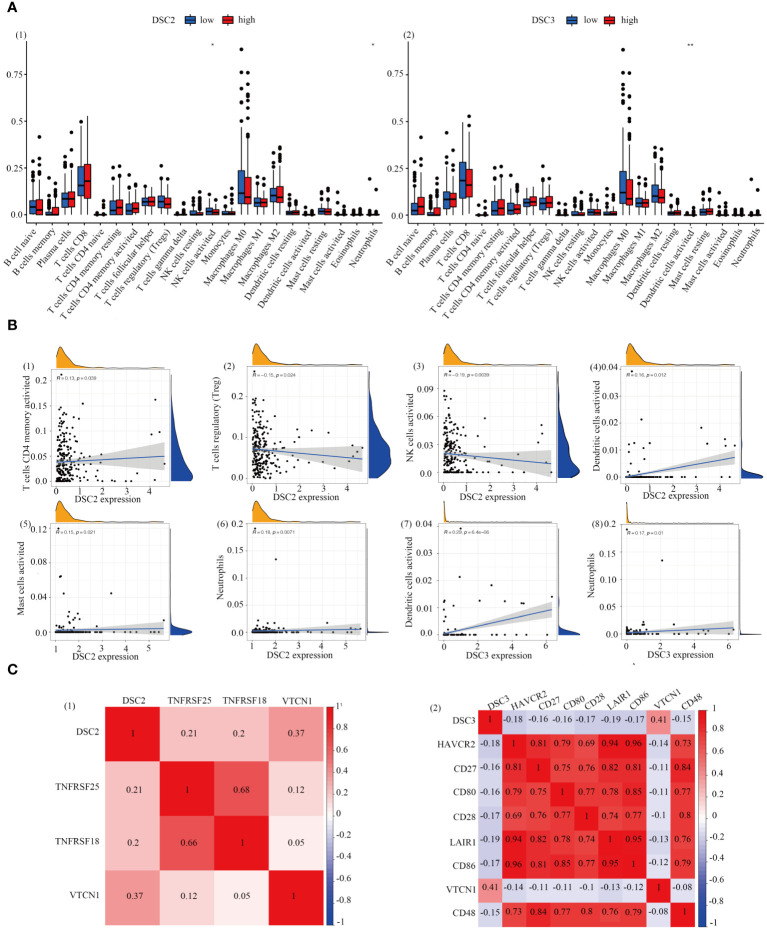
Correlation analysis. **(A)** Assessment of immune infiltration levels based on DSC2 and DSC3 expression groups. **(B)** Evaluation of immune relationship levels within DSC2 and DSC3 expression groups. **(C)** Examination of associations between DSC2, DSC3, and highly correlated immune checkpoint genes. *P < 0.05, **P < 0.01.

Immunohistochemical analysis revealed heightened infiltration levels of NK cells, with significant differences observed between groups with varying DSC2 expression levels. Likewise, significant disparities in dendritic cell activation and infiltration were evident between the two DSC3 expression level groups (**Figure 6A**). Furthermore, a positive correlation was identified between DSC2 expression and activation of CD4 memory T cells, dendritic cells, mast cells, and neutrophil infiltration. Conversely, DSC2 expression exhibited a negative correlation with regulatory T cells and activated NK cell infiltration (**Figure 6B**). Additionally, DSC3 expression showed a positive correlation with dendritic cell activation and neutrophil infiltration. **Figure 6C** depicts the association between DSC2 and DSC3 and highly correlated immune checkpoint genes.

### 3.7 Analysis of GSVA

**Figure 7:**
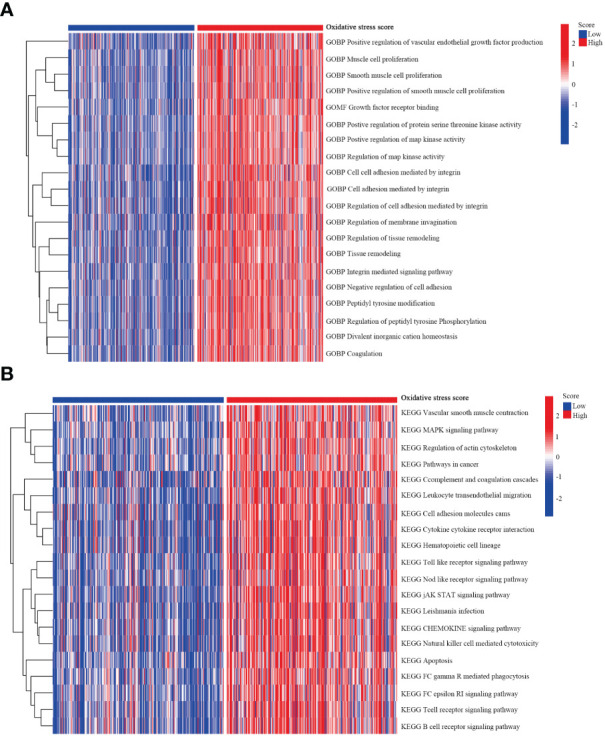
GSVA analysis. **(A)** GSVA depicting the relationship between oxidative stress and KEGG pathways. **(B)** GSVA illustrating the relationship between oxidative stress and GO pathways.

Subsequently, we compared the functional pathways associated with different oxidative stress scores in melanoma. Utilizing GSVA, we assessed the oxidative stress-related signaling pathways in melanoma and further explored them based on KEGG and GO datasets. Enriched genes, signaling pathways, and functions were highlighted using heatmap plots. In the KEGG analysis, we investigated the top 20 associated signaling pathways such as the MAPK signaling pathway, JAK STAT signaling pathway (**Figure 7A**). Additionally, GO analysis revealed significant differential functional pathways between the two oxidative stress groups, presenting the top 20 signals in **Figure 7B**, including muscle cell proliferation, growth factor receptor binding.

### 3.8 Single-cell analysis reveals the expression patterns of oxidative stress in subpopulations of SKMC cells

**Figure 8:**
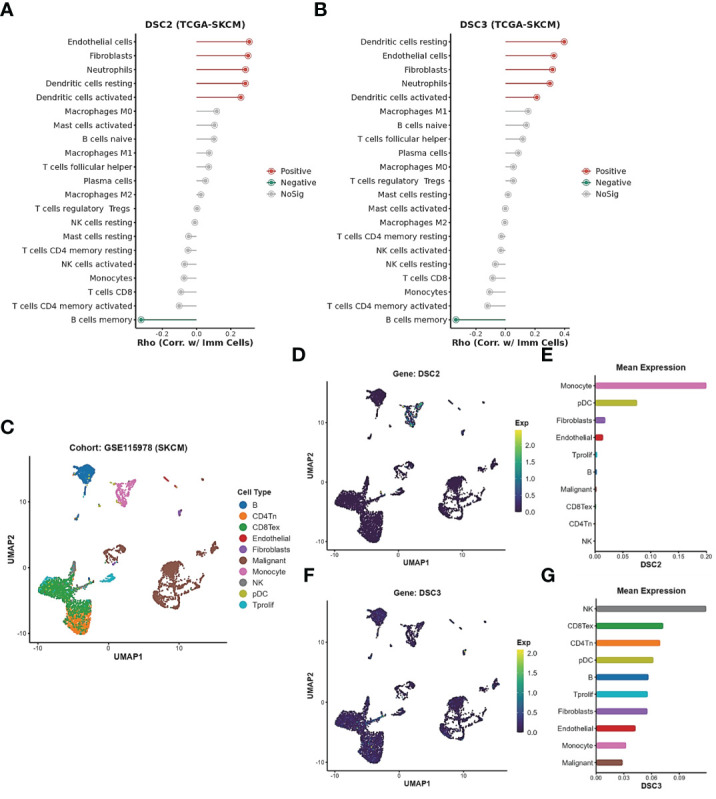
Expression patterns of oxidative stress pathways in single-cell subtypes. **(A, B)** Correlation between DSC2, DSC3, and immune cell infiltration. **(C)** Subtypes of single cells in SKCM patients. **(D, E)** Expression patterns of DSC2 and DSC3 in subtypes of SKCM patient cells. **(F, G)** Expression patterns of DSC3 in subtypes of SKCM patient cells.

We identified a close association between DSC2 and DSC3 with oxidative stress processes. Further investigation demonstrated a positive correlation between the expression of DSC2 and DSC3 and the infiltration of activated dendritic cells and neutrophils, while showing a negative correlation with memory B cells (**Figures 8A, B**). Subsequently, we delved deeper into the expression patterns of DSC2 and DSC3 at the single-cell level (**Figure 8C**). Remarkably, the average expression level of DSC2 was highest in monocytes, followed by pDCs (**Figures 8D, E**). Interestingly, DSC3 was predominantly enriched in NK cells, CD8 T cells, and CD4 T cells (**Figures 8F, G**), indicating distinct spatial distributions of these two oxidative stress molecules.

### 3.9 Impact of DSC2 on immunotherapy response and chemotherapy resistance

**Figure 9:**
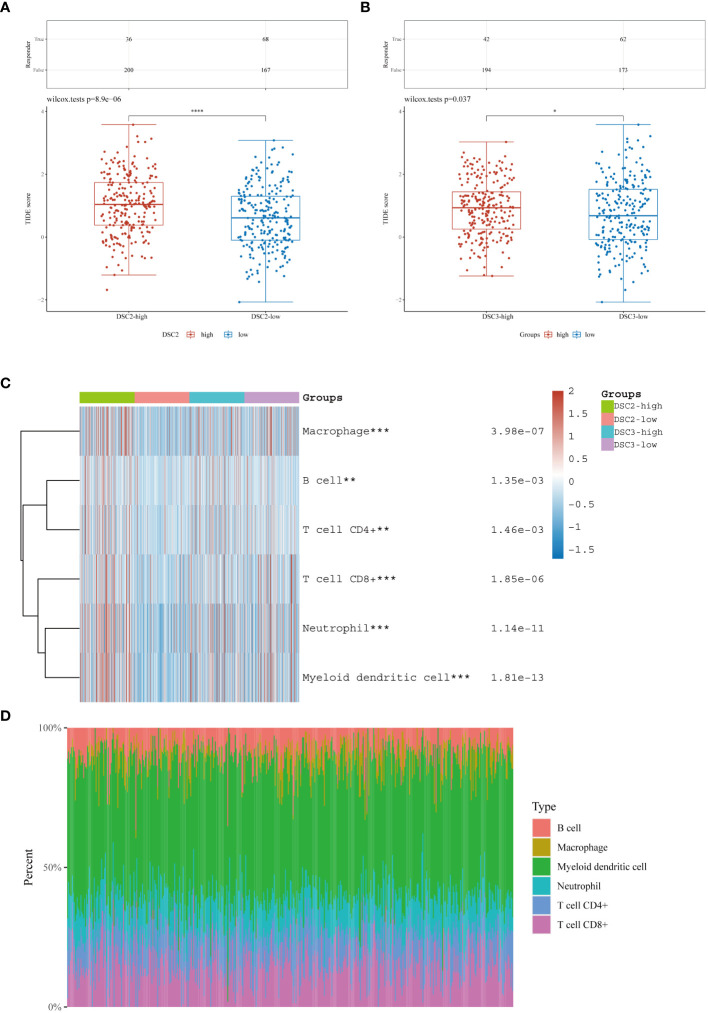
Immune response and infiltration landscape. **(A, B)** Expression levels of oxidative stress pathway genes in immune therapy responders and non-responders among SKCM patients. **(C, D)** Landscape of immune infiltration in SKCM patients with differential expression of oxidative stress pathway genes. *P < 0.05, **P < 0.01, ***P < 0.001, ****P < 0.0001.

**Figure 10:**
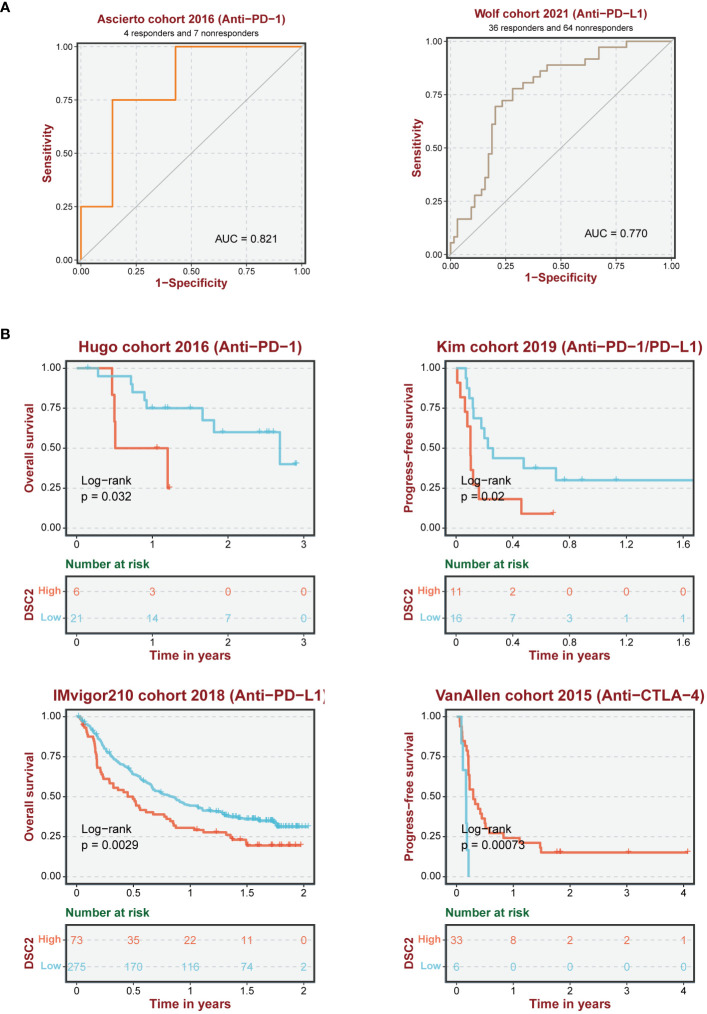
Reliability of immune response prediction. **(A)** ROC curve demonstrating the predictive performance of oxidative stress pathway genes for immune therapy response. **(B)** Survival analysis of SKCM patients receiving immune therapy.

**Figure 11:**
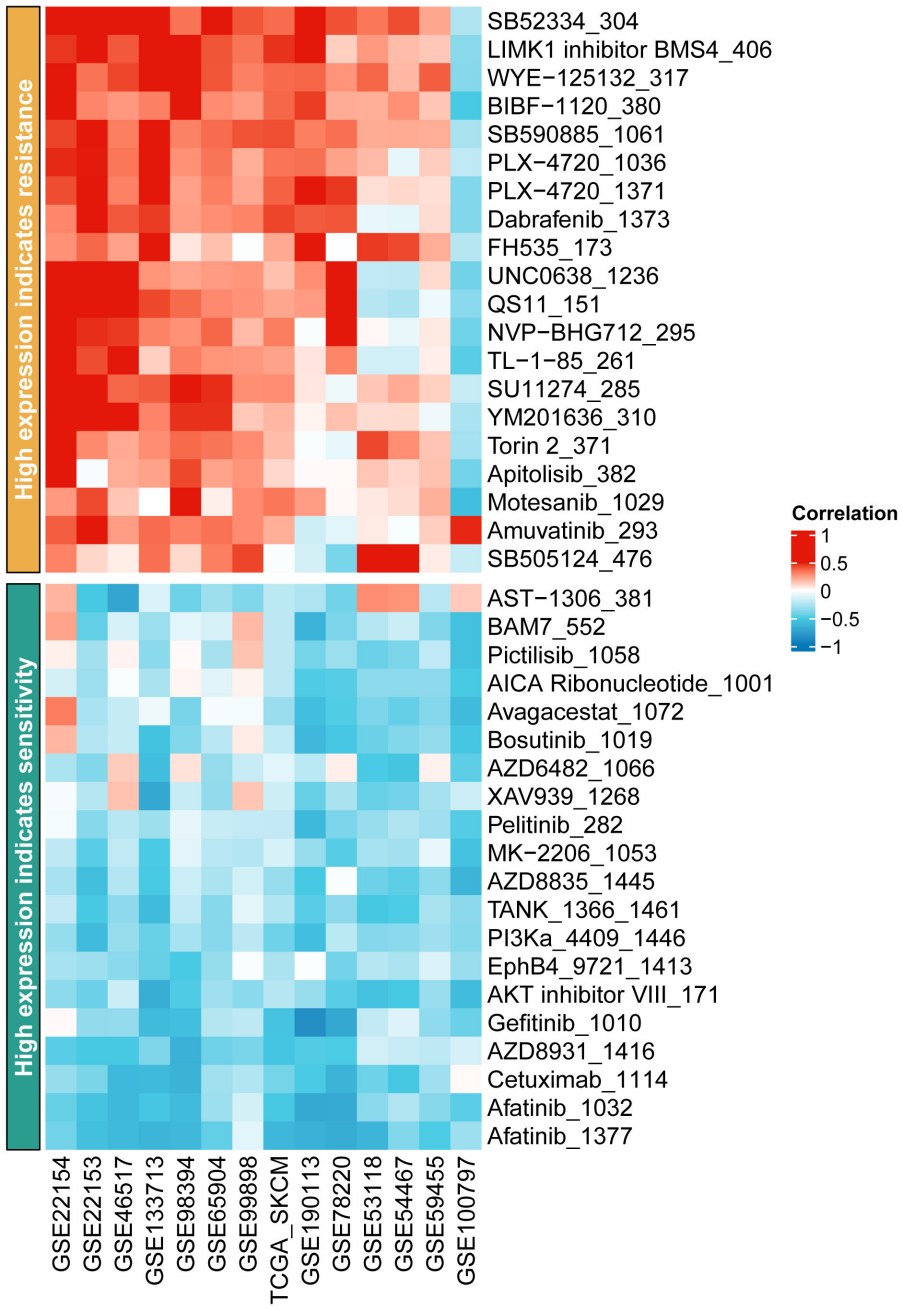
Prediction of drug resistance in subgroups of SKCM patients.

Immunotherapy, as a prominently featured anticancer strategy in recent years, has demonstrated significant efficacy in the treatment of SKCM. However, some patients exhibit insensitivity or even develop resistance to immunotherapy. Thus, we further investigated the role of oxidative stress-related genes in SKCM immunotherapy. It was observed that patients with high expression levels of DSC2 and DSC3 were more sensitive to immunotherapy (**Figures 9A, B**), possibly due to infiltration by macrophages and CD8 T cells (**Figures 9C, D**). Additionally, DSC2 could serve as an effective predictive biomarker for immunotherapy response in SKCM patients (**Figure 10A**). Intriguingly, SKCM patients with high expression of DSC2 exhibited poorer prognosis following Anti-PD-1/PD-L1 therapy compared to those with lower DSC2 expression (**Figure 10B**), which may be attributed to the pro-tumorigenic characteristics of DSC2. Furthermore, chemotherapy, as a crucial component of anticancer therapy, often fails ultimately due to the development of resistance. Therefore, we further investigated the expression of DSC2 in drug resistance. Results indicated that SKCM patients with high expression of DSC2 were more prone to developing resistance to Apitolisib, Motesanib, and Amuvatinib (**Figure 11**), suggesting DSC2 as one of the targeted strategies to enhance chemotherapy sensitivity.

### 3.10 Immunohistochemistry reveals distinct expression patterns of core proteins across trunk subtypes

**Figure 12:**
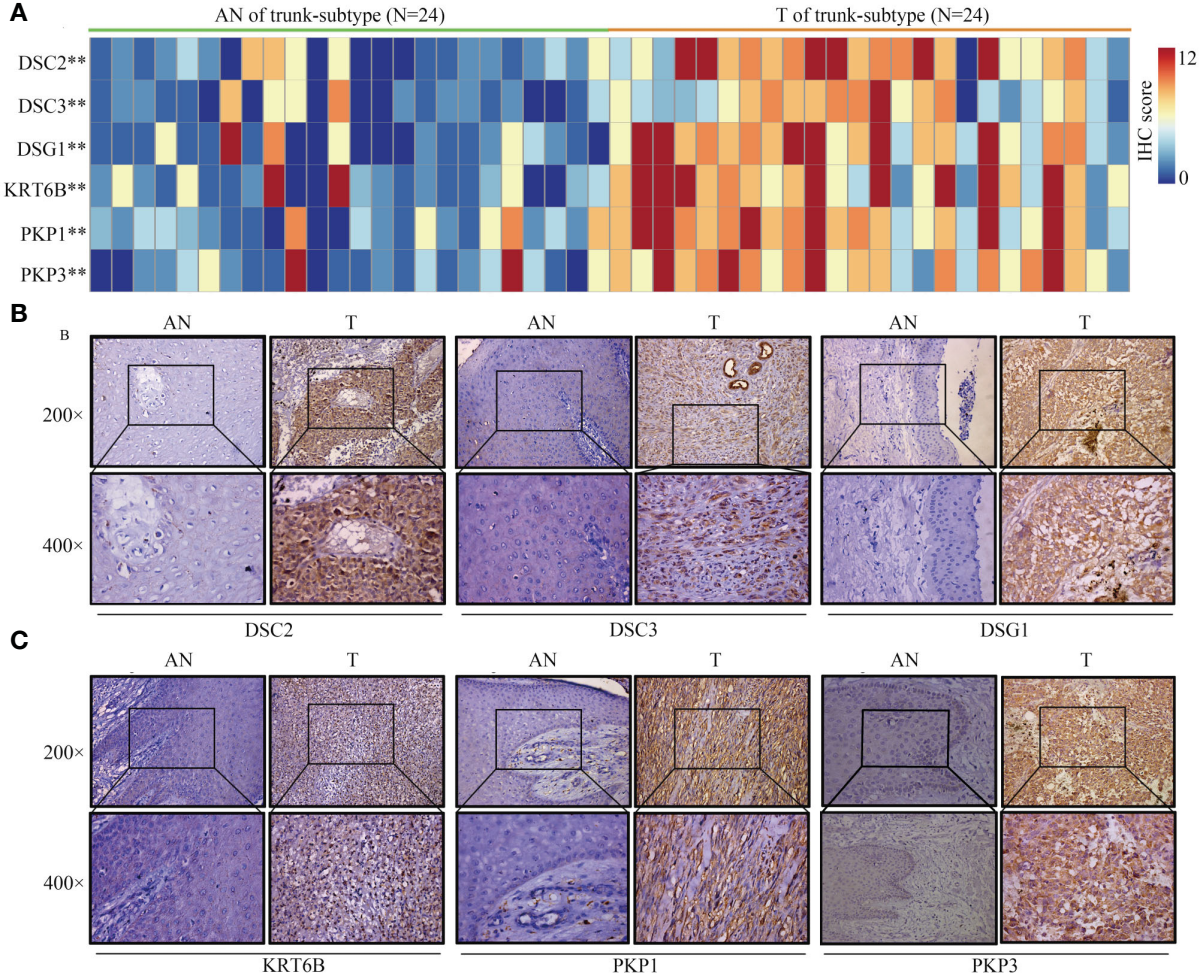
Expression patterns of core proteins in trunk-subtype. **(A)** Heatmap illustrating expression patterns. **(B, C)** Immunohistochemistry confirming high expression of DSC2, DSC3, DSG1, KRT6B, PKP1, and PKP3 in trunk-subtype SKCM. **P < 0.01.

**Figure 13:**
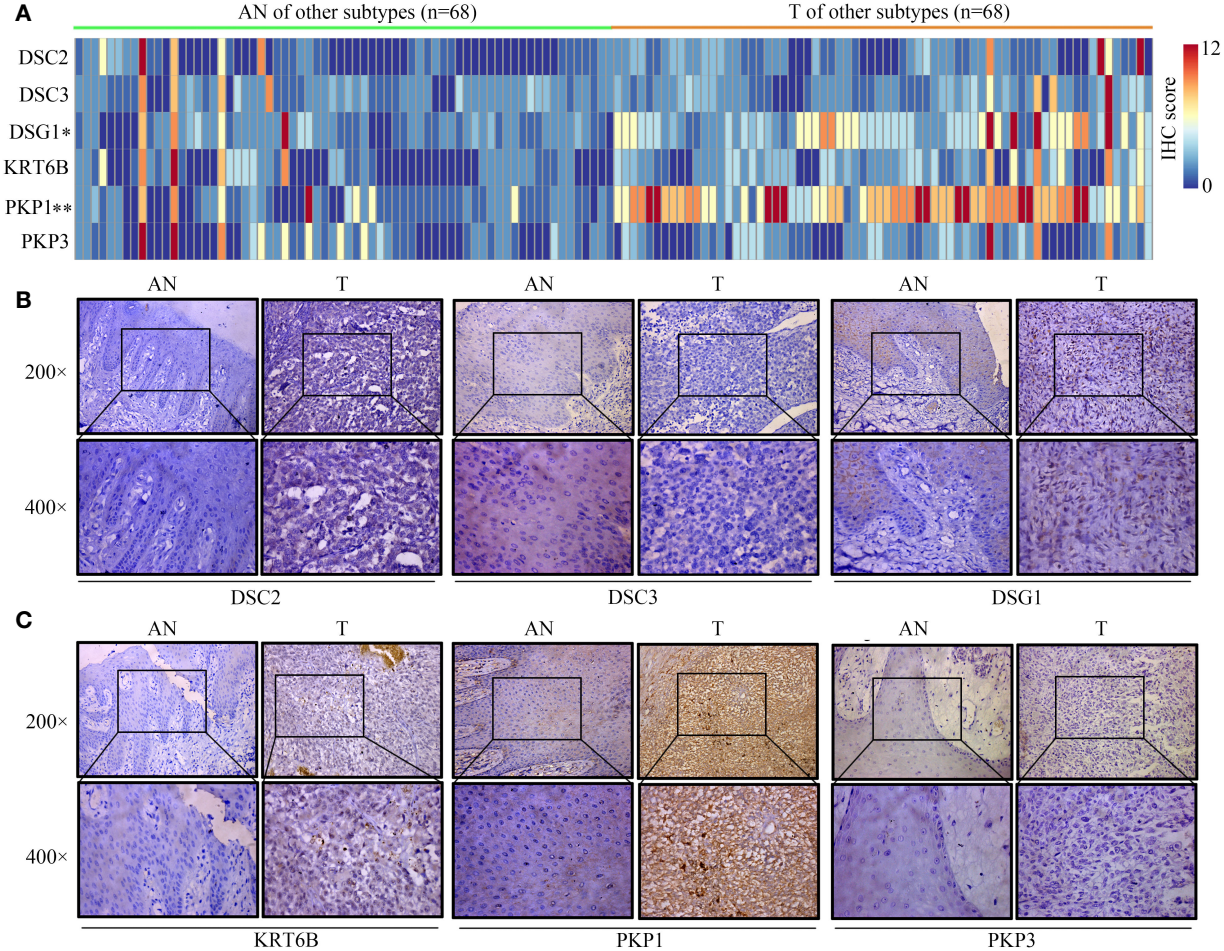
Expression patterns of core proteins in other-subtype. **(A)** Heatmap depicting expression patterns. **(B, C)** Immunohistochemistry confirming the expression of DSC2, DSC3, DSG1 in other-subtype SKCM. *P < 0.05, **P < 0.01.

Given the lack of expression profiles for adjacent normal tissues within the TCGA-SKCM cohort, a total of 92 SKCM samples were obtained, comprising both tumor and adjacent normal tissues. Differential expression analysis of these core proteins was subsequently performed through immunohistochemistry experiments (**Figures 12A**, **13A**). The results indicate that in trunk subtype melanomas, the protein expression levels of DSC2, DSC3, DSG1, KRT6B, PKP1, and PKP3 are significantly higher than those in adjacent normal samples (**Figures 12B, C**), suggesting the crucial roles of these central genes in SKCM progression. However, in other subtype melanoma patients, only DSG1 and PKP1 exhibit significantly elevated protein expression levels compared to adjacent normal samples, with no observed differences in other genes between tumor and adjacent normal samples (**Figures 13B, C**).

## 4 Discussion

SKCM continues to challenge oncology, with its heterogeneity in clinicopathological and cytological features complicating prognosis and treatment (30). Recent advancements have highlighted the role of oxidative stress in melanoma progression, metastasis, and resistance to therapy (31). This study leveraged high-throughput data to unravel the complex interplay between hub genes, oxidative stress, and the immune landscape in SKCM, offering novel insights into its pathogenesis and potential therapeutic targets.

Our analysis, grounded in WGCNA and PPI networks, identified six genes (DSC2, DSC3, DSG1, KRT6B, PKP1, PKP3) with pivotal roles in melanoma’s oxidative stress response and immune infiltration (32, 33). Particularly, DSC2 and DSC3’s association with oxidative stress underscores their potential as biomarkers for SKCM prognosis and therapy response (34). The significant variation in their expression between melanoma subtypes and adjacent normal tissues suggests these genes influence melanoma development, possibly by modulating cell adhesion pathways crucial for tumor metastasis. SKCM can occur on various parts of the human body, indicating potential significant heterogeneity among them. Emi et al., using “anatomical location” as a variable, classified melanoma patients into three categories: limbs, trunk, and head/neck. They discovered that melanomas at different anatomical locations possess distinct risk factors (35). Epidemiological studies have shown that most melanomas are located on the lower limbs, followed by the trunk, with melanomas of the head and neck and those with unspecified anatomical locations having the poorest survival rates (36). Trunk SKCMs tend to have less sun exposure compared to facial SKCMs, and studies have confirmed that anti-PD-1 immunotherapy is particularly recommended for melanomas originating from areas of chronic sun exposure (37). This underscores the necessity of conducting separate research on trunk-SKCM and non-trunk SKCM. Our findings indicate that trunk-SKCMs, compared to normal tissue, exhibit higher activation of oxidative stress pathways, such as DSC2 and DSC3. Conversely, these differences are not significantly observed in other subtypes of SKCM.

In the progression and onset of cancer, the immune system assumes a pivotal role (38, 39), rendering immunotherapy research a promising therapeutic avenue (40, 41). Immune infiltration analysis revealed a significant correlation between the expression levels of DSC2, DSC3, and the infiltration of various immune cells, highlighting the intricate relationship between the tumor microenvironment and melanoma progression (42). High DSC2 expression correlated with increased infiltration of CD4 memory activated T cells, dendritic cells, mast cells, and neutrophils, while negatively correlating with regulatory T cells and activated NK cells. Similar to our study, Li et al. found that the proportion of CD4+ memory-activated T cells is higher in metastatic melanoma. These T cells may become exhausted due to excessive activation, exhibiting tumor immune suppression (17, 43). Early infiltration of mast cells is found in various human and animal tumors, particularly malignant melanoma (44). Within the tumor, mast cells interact with infiltrating immune cells, tumor cells, and ECM through direct cell-cell interactions or by releasing various mediators capable of reshaping the TME (44, 45). Mast cells can facilitate tumor angiogenesis and tumor cell proliferation by releasing pro-angiogenic and pro-tumorigenic factors (46–48). Similarly, DSC3’s expression positively correlated with dendritic cells and neutrophil infiltration (49, 50). Neutrophils play a crucial role in tumor development by promoting tumor cell proliferation, invasion, and metastasis (51, 52). These findings suggest that DSC2 and DSC3 not only contribute to the oxidative stress response but also play a central role in modulating the immune landscape of SKCM.

A multitude of variables exert influence on the progression of tumors (53–56), encompassing proliferation, metastasis (57), and resistance to treatment (58). The role of oxidative stress in tumor immune infiltration represents a focal point of contemporary research interest (59). Excessive oxidative stress can detrimentally impact the immune system. For instance, elevated levels of oxidative stress may precipitate apoptosis or impair functionality in immune cells, thereby diminishing the efficacy of immune infiltration (60–62). Moreover, oxidative stress may facilitate the proliferation and functional enhancement of immune suppressive cells within the tumor microenvironment, such as TAMs, further inhibiting the immune cells’ action against tumors (63, 64). Currently, immunotherapy is recognized as an effective treatment strategy for various cancers (65). Yu et al. have delineated the interactions between oxidative stress and the TME suggesting the potential of oxidative stress to augment immunotherapy (66). Our findings indicate that high expression of DSC2 and DSC3 is indicative of an improved immune response (**Figures 9A, B**). However, this is inversely related to survival in patients undergoing anti-PD-1/PD-L1 therapy, while positively correlated with survival benefits in patients treated with anti-CTLA-4 therapy (**Figure 10B**). This suggests that the oxidative stress pathway may influence the expression patterns of CTLA-4.

Moreover, our study delves into the impact of these genes on drug resistance and response to immunotherapy (67, 68). The expression of DSC2, in particular, was found to influence SKCM patients’ sensitivity to immunotherapy and chemotherapy drugs, pointing toward its potential as a predictive biomarker for treatment response (69). This is critical, considering the increasing application of immunotherapy in SKCM treatment and the challenge of overcoming drug resistance. It is worth noting that Sven et al. found that knockdown of DSC2 in mice resulted in damaged colonic mucosal repair, which was associated with decreased levels of integrins β1 and β4 (70, 71). Additionally, Vite et al. observed that low expression of DSC2 was associated with ventricular developmental abnormalities (72). Therefore, in future studies targeting DSC2 for the treatment of SKCM, it is necessary to consider its role in protecting myocardium and intestinal mucosa function.

However, this study is not without limitations. The reliance on TCGA data, with its scarce normal samples, introduces potential biases (73), and the six hub genes identified may not cover all genes associated with SKCM survival (74). Furthermore, our conclusions are primarily drawn from bioinformatic analyses and require validation through experimental studies and prospective cohorts to confirm these genes’ roles in SKCM pathogenesis and their therapeutic potential.

In conclusion, this investigation enriches our understanding of melanoma’s molecular underpinnings, emphasizing the significance of oxidative stress and immune infiltration in its progression. The identified hub genes offer promising directions for future research into targeted therapies for SKCM, aiming to improve patient prognosis and combat resistance to existing treatments. Further experimental validation and clinical trials are essential to translate these findings into clinical applications, potentially revolutionizing melanoma treatment strategies.

## Data availability statement

The original contributions presented in the study are included in the article/supplementary material. Further inquiries can be directed to the corresponding authors.

## Ethics statement

The studies involving humans were approved by Guizhou Medical University Ethics Committee. The studies were conducted in accordance with the local legislation and institutional requirements. The participants provided their written informed consent to participate in this study.

## Author contributions

DR: Data curation, Formal analysis, Funding acquisition, Methodology, Project administration, Software, Validation, Visualization, Writing – original draft. YS: Data curation, Formal analysis, Visualization, Writing – original draft. DJ:Investigation, Resources, Visualization, Writing – original draft. ZZ: Resources, Visualization, Writing – original draft. YY: Data curation, Investigation, Resources, Writing – original draft. HL: Conceptualization, Investigation, Methodology, Project administration, Validation, Writing – review & editing. DW:Conceptualization, Investigation, Supervision, Validation, Writing – review & editing. YC: Conceptualization, Investigation, Methodology, Project administration, Resources, Validation, Writing – review & editing, Funding acquisition.

## References

[1] Lopes F Sleiman MG Sebastian K Bogucka R Jacobs EA Adamson AS. UV exposure and the risk of cutaneous melanoma in skin of color: A systematic review. JAMA Dermatol. (2021) 157:213–9. doi: 10.1001/jamadermatol.2020.4616 33325988

[2] Sun L Guan Z Wei S Tan R Li P Yan L. Identification of long non-coding and messenger RNAs differentially expressed between primary and metastatic melanoma. Front Genet. (2019) 10:292. doi: 10.3389/fgene.2019.00292 31024618 PMC6459964

[3] Wang X Xiong H Liang D Chen Z Li X Zhang K. The role of SRGN in the survival and immune infiltrates of skin cutaneous melanoma (SKCM) and SKCM-metastasis patients. BMC Cancer. (2020) 20:378. doi: 10.1186/s12885-020-06849-7 32370744 PMC7201763

[4] Zhang S Chen K Zhao Z Zhang X Xu L Liu T. Lower expression of GBP2 associated with less immune cell infiltration and poor prognosis in skin cutaneous melanoma (SKCM). J Immunother. (2022) 45:274–83. doi: 10.1097/CJI.0000000000000421 35543550

[5] Xiong J Chi H Yang G Zhao S Zhang J Tran LJ. Revolutionizing anti-tumor therapy: unleashing the potential of B cell-derived exosomes. Front Immunol. (2023) 14:1188760. doi: 10.3389/fimmu.2023.1188760 37342327 PMC10277631

[6] Wang J Zuo Z Yu Z Chen Z Meng X Ma Z. et al: Single-cell transcriptome analysis revealing the intratumoral heterogeneity of ccRCC and validation of MT2A in pathogenesis. Funct Integr Genomics. (2023) 23:300. doi: 10.1007/s10142-023-01225-7 37713131

[7] Selitsky SR Mose LE Smith CC Chai S Hoadley KA Dittmer DP. Prognostic value of B cells in cutaneous melanoma. Genome Med. (2019) 11:36. doi: 10.1186/s13073-019-0647-5 31138334 PMC6540526

[8] Piskounova E Agathocleous M Murphy MM Hu Z Huddlestun SE Zhao Z. Oxidative stress inhibits distant metastasis by human melanoma cells. Nature. (2015) 527:186–91. doi: 10.1038/nature15726 PMC4644103 26466563

[9] Song G Peng G Zhang J Song B Yang J Xie X. Uncovering the potential role of oxidative stress in the development of periodontitis and establishing a stable diagnostic model via combining single-cell and machine learning analysis. Front Immunol. (2023) 14:1181467. doi: 10.3389/fimmu.2023.1181467 37475857 PMC10355807

[10] Pilco-Ferreto N Calaf GM. Influence of doxorubicin on apoptosis and oxidative stress in breast cancer cell lines. Int J Oncol. (2016) 49:753–62. doi: 10.3892/ijo.2016.3558 27278553

[11] Hu H Yang W Liang Z Zhou Z Song Q Liu W. et al: Amplification of oxidative stress with lycorine and gold-based nanocomposites for synergistic cascade cancer therapy. J Nanobiotechnology. (2021) 19:221. doi: 10.1186/s12951-021-00933-1 34315494 PMC8314456

[12] Srivastava AK Wang Y Huang R Skinner C Thompson T Pollard L. Human genome meeting 2016 : Houston, TX, USA. 28 February - 2 March 2016. Hum Genomics. (2016) 10:12. doi: 10.1186/s40246-016-0063-5 27294413 PMC4896275

[13] Zhao S Chi H Yang Q Chen S Wu C Lai G. et al: Identification and validation of neurotrophic factor-related gene signatures in glioblastoma and Parkinson's disease. Front Immunol. (2023) 14:1090040. doi: 10.3389/fimmu.2023.1090040 36825022 PMC9941742

[14] Chi H Yang J Peng G Zhang J Song G Xie X. Circadian rhythm-related genes index: A predictor for HNSCC prognosis, immunotherapy efficacy, and chemosensitivity. Front Immunol. (2023) 14:1091218. doi: 10.3389/fimmu.2023.1091218 36969232 PMC10036372

[15] Zhao S Zhang X Gao F Chi H Zhang J Xia Z. Identification of copper metabolism-related subtypes and establishment of the prognostic model in ovarian cancer. Front Endocrinol (Lausanne). (2023) 14:1145797. doi: 10.3389/fendo.2023.1145797 36950684 PMC10025496

[16] Ren Q Zhang P Lin H Feng Y Chi H Zhang X. A novel signature predicts prognosis and immunotherapy in lung adenocarcinoma based on cancer-associated fibroblasts. Front Immunol. (2023) 14:1201573. doi: 10.3389/fimmu.2023.1201573 37325647 PMC10264584

[17] Chi H Zhao S Yang J Gao X Peng G Zhang J. et al: T-cell exhaustion signatures characterize the immune landscape and predict HCC prognosis via integrating single-cell RNA-seq and bulk RNA-sequencing. Front Immunol. (2023) 14:1137025. doi: 10.3389/fimmu.2023.1137025 37006257 PMC10050519

[18] Liu J Zhang P Yang F Jiang K Sun S Xia Z. Integrating single-cell analysis and machine learning to create glycosylation-based gene signature for prognostic prediction of uveal melanoma. Front Endocrinol (Lausanne). (2023) 14:1163046. doi: 10.3389/fendo.2023.1163046 37033251 PMC10076776

[19] Zhang J Peng G Chi H Yang J Xie X Song G. CD8 + T-cell marker genes reveal different immune subtypes of oral lichen planus by integrating single-cell RNA-seq and bulk RNA-sequencing. BMC Oral Health. (2023) 23:464. doi: 10.1186/s12903-023-03138-0 37422617 PMC10329325

[20] Zhang P Pei S Wu L Xia Z Wang Q Huang X. Integrating multiple machine learning methods to construct glutamine metabolism-related signatures in lung adenocarcinoma. Front Endocrinol (Lausanne). (2023) 14:1196372. doi: 10.3389/fendo.2023.1196372 37265698 PMC10229769

[21] Chen X Jiang X Wang H Wang C Wang C Pan C. et al: DNA methylation-regulated SNX20 overexpression correlates with poor prognosis, immune cell infiltration, and low-grade glioma progression. Aging (Albany NY). (2022) 14:5211–22. doi: 10.18632/aging.v14i12 PMC9271302 35771139

[22] Chi H Gao X Xia Z Yu W Yin X Pan Y. et al: FAM family gene prediction model reveals heterogeneity, stemness and immune microenvironment of UCEC. Front Mol Biosci. (2023) 10:1200335. doi: 10.3389/fmolb.2023.1200335 37275958 PMC10235772

[23] Zhang X Zhuge J Liu J Xia Z Wang H Gao Q. et al: Prognostic signatures of sphingolipids: Understanding the immune landscape and predictive role in immunotherapy response and outcomes of hepatocellular carcinoma. Front Immunol. (2023) 14:1153423. doi: 10.3389/fimmu.2023.1153423 37006285 PMC10063861

[24] Chi H Xie X Yan Y Peng G Strohmer DF Lai G. Natural killer cell-related prognosis signature characterizes immune landscape and predicts prognosis of HNSCC. Front Immunol. (2022) 13:1018685. doi: 10.3389/fimmu.2022.1018685 36263048 PMC9575041

[25] Xia Z Chen S He M Li B Deng Y Yi L. Editorial: Targeting metabolism to activate T cells and enhance the efficacy of checkpoint blockade immunotherapy in solid tumors. Front Immunol. (2023) 14:1247178. doi: 10.3389/fimmu.2023.1247178 37575246 PMC10415066

[26] Wang X Zhao Y Strohmer DF Yang W Xia Z Yu C. The prognostic value of MicroRNAs associated with fatty acid metabolism in head and neck squamous cell carcinoma. Front Genet. (2022) 13:983672. doi: 10.3389/fgene.2022.983672 36110217 PMC9468645

[27] Chi H Jiang P Xu K Zhao Y Song B Peng G. A novel anoikis-related gene signature predicts prognosis in patients with head and neck squamous cell carcinoma and reveals immune infiltration. Front Genet. (2022) 13:984273. doi: 10.3389/fgene.2022.984273 36092898 PMC9459093

[28] Goeman JJ van de Geer SA de Kort F van Houwelingen HC. A global test for groups of genes: testing association with a clinical outcome. Bioinformatics. (2004) 20:93–9. doi: 10.1093/bioinformatics/btg382 14693814

[29] Xiao J Lin H Liu B Xia Z Zhang J Jin J. Decreased S1P and SPHK2 are involved in pancreatic acinar cell injury. biomark Med. (2019) 13:627–37. doi: 10.2217/bmm-2018-0404 31157539

[30] Cabrera R Recule F. Unusual clinical presentations of Malignant melanoma: A review of clinical and histologic features with special emphasis on dermatoscopic findings. Am J Clin Dermatol. (2018) 19:15–23. doi: 10.1007/s40257-018-0373-6 PMC6244635 30374898

[31] Yu X Cong P Wei W Zhou Y Bao Z Hou H. Construction of prognostic risk model of patients with skin cutaneous melanoma based on TCGA-SKCM methylation cohort. Comput Math Methods Med. (2022) 2022:4261329. doi: 10.1155/2022/4261329 36060650 PMC9436567

[32] Han Y Li X Yan J Ma C Wang X Pan H. Bioinformatic analysis identifies potential key genes in the pathogenesis of melanoma. Front Oncol. (2020) 10:581985. doi: 10.3389/fonc.2020.581985 33178610 PMC7596746

[33] Sheng Z Han W Huang B Shen G. Screening and identification of potential prognostic biomarkers in metastatic skin cutaneous melanoma by bioinformatics analysis. J Cell Mol Med. (2020) 24:11613–8. doi: 10.1111/jcmm.15822 PMC7576265 32869947

[34] Muller L Rietscher K Keil R Neuholz M Hatzfeld M. Plakophilin 3 phosphorylation by ribosomal S6 kinases supports desmosome assembly. J Cell Sci. (2020) 133:238295. doi: 10.1242/jcs.238295 32122945

[35] Dika E Lambertini M Venturi F Veronesi G Mastroeni S Hrvatin Stancic B. A comparative demographic study of atypical spitz nevi and Malignant melanoma. Acta Dermatovenerol Croat. (2023) 31:165–8. 38439731

[36] de Vries E Uribe C Beltran Rodriguez CC Caparros A Meza E Gil F. Descriptive epidemiology of melanoma diagnosed between 2010 and 2014 in a Colombian cancer registry and a call for improving available data on melanoma in Latin America. Cancers (Basel). (2023) 15:5848. doi: 10.3390/cancers15245848 38136393 PMC10741499

[37] Russo D Dalle S Dereure O Mortier L Dalac-Rat S Dutriaux C. Differential gradients of immunotherapy vs targeted therapy efficacy according to the sun-exposure pattern of the site of occurrence of primary melanoma: a multicenter prospective cohort study (MelBase). Front Oncol. (2023) 13:1250026. doi: 10.3389/fonc.2023.1250026 37936607 PMC10627180

[38] Zhao Y Wei K Chi H Xia Z Li X. IL-7: A promising adjuvant ensuring effective T cell responses and memory in combination with cancer vaccines? Front Immunol. (2022) 13:1022808. doi: 10.3389/fimmu.2022.1022808 36389666 PMC9650235

[39] Gong X Chi H Strohmer DF Teichmann AT Xia Z Wang Q. Exosomes: A potential tool for immunotherapy of ovarian cancer. Front Immunol. (2022) 13:1089410. doi: 10.3389/fimmu.2022.1089410 36741380 PMC9889675

[40] Jin W Yang Q Chi H Wei K Zhang P Zhao G. Ensemble deep learning enhanced with self-attention for predicting immunotherapeutic responses to cancers. Front Immunol. (2022) 13:1025330. doi: 10.3389/fimmu.2022.1025330 36532083 PMC9751999

[41] Xiao J Huang K Lin H Xia Z Zhang J Li D. Mogroside II(E) inhibits digestive enzymes via suppression of interleukin 9/interleukin 9 receptor signalling in acute pancreatitis. Front Pharmacol. (2020) 11:859. doi: 10.3389/fphar.2020.00859 32587518 PMC7298197

[42] Wang F Chen S Liu HB Parent CA Coulombe PA. Keratin 6 regulates collective keratinocyte migration by altering cell-cell and cell-matrix adhesion. J Cell Biol. (2018) 217:4314–30. doi: 10.1083/jcb.201712130 PMC6279382 30389720

[43] Li M Zhao J Yang R Cai R Liu X Xie J. CENPF as an independent prognostic and metastasis biomarker corresponding to CD4+ memory T cells in cutaneous melanoma. Cancer Sci. (2022) 113:1220–34. doi: 10.1111/cas.15303 PMC8990861 35189004

[44] Komi DEA Redegeld FA. Role of mast cells in shaping the tumor microenvironment. Clin Rev Allergy Immunol. (2020) 58:313–25. doi: 10.1007/s12016-019-08753-w PMC7244463 31256327

[45] He Y Wu S Yuan Y Sun Y Ai Q Zhou R. Remodeling tumor immunosuppression with molecularly imprinted nanoparticles to enhance immunogenic cell death for cancer immunotherapy. J Control Release. (2023) 362:44–57. doi: 10.1016/j.jconrel.2023.08.026 37579978

[46] Ribatti D Tamma R Vacca A. Mast cells and angiogenesis in human plasma cell Malignancies. Int J Mol Sci. (2019) 20:481. doi: 10.3390/ijms20030481 30678047 PMC6386864

[47] Xu W Qian J Zeng F Li S Guo W Chen L. Protein kinase Ds promote tumor angiogenesis through mast cell recruitment and expression of angiogenic factors in prostate cancer microenvironment. J Exp Clin Cancer Res. (2019) 38:114. doi: 10.1186/s13046-019-1118-y 30841931 PMC6404326

[48] Sun Z Wang J Zhang Q Meng X Ma Z Niu J. et al: Coordinating single-cell and bulk RNA-seq in deciphering the intratumoral immune landscape and prognostic stratification of prostate cancer patients. Environ Toxicol. (2024) 39:657–68. doi: 10.1002/tox.23928 37565774

[49] Brodehl A Stanasiuk C Anselmetti D Gummert J Milting H. Incorporation of desmocollin-2 into the plasma membrane requires N-glycosylation at multiple sites. FEBS Open Bio. (2019) 9:996–1007. doi: 10.1002/2211-5463.12631 PMC6487837 30942563

[50] Wanuske MT Brantschen D Schinner C Studle C Walter E Hiermaier M. Clustering of desmosomal cadherins by desmoplakin is essential for cell-cell adhesion. Acta Physiol (Oxf). (2021) 231:e13609. doi: 10.1111/apha.13609 33354837

[51] Giese MA Hind LE Huttenlocher A. Neutrophil plasticity in the tumor microenvironment. Blood. (2019) 133:2159–67. doi: 10.1182/blood-2018-11-844548 PMC6524564 30898857

[52] Masucci MT Minopoli M Del Vecchio S Carriero MV. The emerging role of neutrophil extracellular traps (NETs) in tumor progression and metastasis. Front Immunol. (2020) 11:1749. doi: 10.3389/fimmu.2020.01749 33042107 PMC7524869

[53] Zhang L Chen C Chai D Li C Qiu Z Kuang T. Characterization of the intestinal fungal microbiome in patients with hepatocellular carcinoma. J Transl Med. (2023) 21:126. doi: 10.1186/s12967-023-03940-y 36793057 PMC9933289

[54] Zhang H Zhai X Liu Y Xia Z Xia T Du G. et al: NOP2-mediated m5C Modification of c-Myc in an EIF3A-Dependent Manner to Reprogram Glucose Metabolism and Promote Hepatocellular Carcinoma Progression. Res (Wash D C). (2023) 6:0184. doi: 10.34133/research.0184 PMC10313139 37398932

[55] Gong X Chi H Xia Z Yang G Tian G. Advances in HPV-associated tumor management: Therapeutic strategies and emerging insights. J Med Virol. (2023) 95:e28950. doi: 10.1002/jmv.28950 37465863

[56] Soltani M Zhao Y Xia Z Ganjalikhani Hakemi M Bazhin AV. The importance of cellular metabolic pathways in pathogenesis and selective treatments of hematological Malignancies. Front Oncol. (2021) 11:767026. doi: 10.3389/fonc.2021.767026 34868994 PMC8636012

[57] Li Z Zhou H Xia Z Xia T Du G Franziska SD. HMGA1 augments palbociclib efficacy via PI3K/mTOR signaling in intrahepatic cholangiocarcinoma. biomark Res. (2023) 11:33. doi: 10.1186/s40364-023-00473-w 36978140 PMC10053751

[58] Zhai X Xia Z Du G Zhang X Xia T Ma D. LRP1B suppresses HCC progression through the NCSTN/PI3K/AKT signaling axis and affects doxorubicin resistance. Genes Dis. (2023) 10:2082–96. doi: 10.1016/j.gendis.2022.10.021 PMC10363646 37492741

[59] Caliri AW Tommasi S Besaratinia A. Relationships among smoking, oxidative stress, inflammation, macromolecular damage, and cancer. Mutat Res Rev Mutat Res. (2021) 787:108365. doi: 10.1016/j.mrrev.2021.108365 34083039 PMC8287787

[60] Costanzo-Garvey DL Case AJ Watson GF Alsamraae M Chatterjee A Oberley-Deegan RE. Prostate cancer addiction to oxidative stress defines sensitivity to anti-tumor neutrophils. Clin Exp Metastasis. (2022) 39:641–59. doi: 10.1007/s10585-022-10170-x PMC9338904 35604506

[61] Batog G Doloto A Bak E Piatkowska-Chmiel I Krawiec P Pac-Kozuchowska E. The interplay of oxidative stress and immune dysfunction in Hashimoto's thyroiditis and polycystic ovary syndrome: a comprehensive review. Front Immunol. (2023) 14:1211231. doi: 10.3389/fimmu.2023.1211231 37588599 PMC10426741

[62] Dharshini LCP Rasmi RR Kathirvelan C Kumar KM Saradhadevi KM Sakthivel KM. Regulatory components of oxidative stress and inflammation and their complex interplay in carcinogenesis. Appl Biochem Biotechnol. (2023) 195:2893–916. doi: 10.1007/s12010-022-04266-z 36441404

[63] Gonzalez MA Lu DR Yousefi M Kroll A Lo CH Briseno CG. et al: Phagocytosis increases an oxidative metabolic and immune suppressive signature in tumor macrophages. J Exp Med. (2023) 220:20221472. doi: 10.1084/jem.20221472 PMC10067971 36995340

[64] Wu X Lu W Xu C Jiang C Zhuo Z Wang R. et al: macrophages phenotype regulated by IL-6 are associated with the prognosis of platinum-resistant serous ovarian cancer: integrated analysis of clinical trial and omics. J Immunol Res. (2023) 2023:6455704. doi: 10.1155/2023/6455704 37124547 PMC10132904

[65] Yang H Li C Xie QJCI. Advances in the use of nanomaterials in tumour therapy: challenges and prospects. Cancer insight (2023) 2:80–101. doi: 10.58567/ci02010006

[66] Yu Y Wu Y Zhang Y Lu M Su X. Oxidative stress in the tumor microenvironment in gastric cancer and its potential role in immunotherapy. FEBS Open Bio. (2023) 13:1238–52. doi: 10.1002/2211-5463.13630 PMC10315728 37171226

[67] Pizzimenti S Ribero S Cucci MA Grattarola M Monge C Dianzani C. Oxidative stress-related mechanisms in melanoma and in the acquired resistance to targeted therapies. Antioxidants (Basel). (2021) 10:1942. doi: 10.3390/antiox10121942 34943045 PMC8750393

[68] Yang H Li Z Zhu S Wang W Zhang J Zhao D. Molecular mechanisms of pancreatic cancer liver metastasis: the role of PAK2. Front Immunol. (2024) 15:1347683. doi: 10.3389/fimmu.2024.1347683 38343537 PMC10853442

[69] Gagliano N Donne ID Torri C Migliori M Grizzi F Milzani A. et al: Early cytotoxic effects of ochratoxin A in rat liver: a morphological, biochemical and molecular study. Toxicology. (2006) 225:214–24. doi: 10.1016/j.tox.2006.06.004 16857307

[70] Flemming S Luissint AC Kusters DHM Raya-Sandino A Fan S Zhou DW. Desmocollin-2 promotes intestinal mucosal repair by controlling integrin-dependent cell adhesion and migration. Mol Biol Cell. (2020) 31:407–18. doi: 10.1091/mbc.E19-12-0692 PMC7185897 31967937

[71] Raya-Sandino A Luissint AC Kusters DHM Narayanan V Flemming S Garcia-Hernandez V. Regulation of intestinal epithelial intercellular adhesion and barrier function by desmosomal cadherin desmocollin-2. Mol Biol Cell. (2021) 32:753–68. doi: 10.1091/mbc.E20-12-0775 PMC8108520 33596089

[72] Vite A Gandjbakhch E Prost C Fressart V Fouret P Neyroud N. Desmosomal cadherins are decreased in explanted arrhythmogenic right ventricular dysplasia/cardiomyopathy patient hearts. PloS One. (2013) 8:e75082. doi: 10.1371/journal.pone.0075082 24086444 PMC3781033

[73] Jiang S Yang X Lin Y Liu Y Tran LJ Zhang J. Unveiling Anoikis-related genes: A breakthrough in the prognosis of bladder cancer. J Gene Med. (2024) 26:e3651. doi: 10.1002/jgm.3651 38282152

[74] Kurinna S Schafer M Ostano P Karouzakis E Chiorino G Bloch W. A novel Nrf2-miR-29-desmocollin-2 axis regulates desmosome function in keratinocytes. Nat Commun. (2014) 5:5099. doi: 10.1038/ncomms6099 25283360

